# Heterogeneity by age and gender in the association of kidney function with mortality among patients with diabetes - analysis of diabetes registry in Singapore

**DOI:** 10.1186/s12882-024-03463-8

**Published:** 2024-01-17

**Authors:** Tazeen H. Jafar, Loraine Liping Seng, Yeli Wang, Ching Wee Lim, Choong Meng Chan, Jia Liang Kwek, Thomas M. Coffman, Yongjing Ping, Yong Mong Bee, John C. Allen

**Affiliations:** 1 Program in Health Services and Systems Research, Duke-NUS Medical School, 8 College Road, Singapore, 169857 Singapore; 2 Department of Renal Medicine, Singapore General Hospital, Singapore, Singapore; 3 Duke Global Health Institute, Duke University, Durham, NC USA; 4 Nutrition Department, Harvard T.H. Chan School of Public Health, Boston, MA USA; 5 Cardiovascular & Metabolic Disorders Programme, Duke-NUS Medical School, Singapore, Singapore; 6 Department of Medicine, Duke University School of Medicine, Durham, NC USA; 7 Department of Endocrinology, Singapore General Hospital, Singapore, Singapore; 8 Center for Quantitative Medicine, Office of Clinical Sciences, Duke-NUS Medical School, Singapore, Singapore

**Keywords:** Gender, Age, Disparity, Death, Estimated glomerular filtration rate

## Abstract

**Background:**

We aimed to explore the three-way interaction among age, gender, and kidney function on the risk of all-cause mortality and cardiovascular mortality among patients with type 2 diabetes (T2D).

**Methods:**

In a retrospective cohort study, patients aged > 40 years with T2D with serum creatinine and urine albumin measured from 2013 to 2019 were included from a multi-institutional diabetes registry. The exposure was estimated glomerular filtration rate (eGFR), outcomes were all-cause mortality (primary outcome) and cardiovascular disease (CVD) mortality (secondary outcome). We applied multivariable cox proportional hazards regression analysis to compute the association between eGFR and mortality.

**Results:**

A total of 36,556 patients were followed for up to 6 years during which 2492 (6.82%) died from all causes, and 690 (1.9%) died from CVD. We observed a significant three-way interaction (*p* = 0.021) among age (younger, < 65; older, ≥65 years), gender and eGFR for the risk of all-cause mortality. Using age- and gender-specific eGFR of 90 ml/min/1.73m^2^ as the reference point, the adjusted hazard rate (HR) (95% CI) for all-cause mortality at eGFR of 40 ml/min/1.73m^2^ was 3.70 (2.29 to 5.99) in younger women and 1.86 (1.08 to 3.19) in younger men. The corresponding adjusted HRs in older women and older men were 2.38 (2.02 to 2.82) and 2.18 (1.85 to 2.57), respectively. Similar results were observed for CVD deaths, although the three-way interaction was not statistically significant. Sensitivity analysis yielded similar results.

**Conclusions:**

In this T2D population, younger women with reduced kidney function might be more susceptible to higher risks of all-cause mortality and CVD mortality than younger men.

**Supplementary Information:**

The online version contains supplementary material available at 10.1186/s12882-024-03463-8.

## Introduction

Diabetes is a major public health problem globally [1]. Reduced kidney function is an established risk factor for all-cause deaths, cardiovascular disease (CVD) deaths, and end-stage kidney disease (ESKD)—with no exception for individuals with diabetes [2, 3]. Several studies and a meta-analysis of individual level data associate declines in estimated glomerular filtration rate (eGFR) below a threshold of about 75 ml/min/1.73m^2^ with increases in all-cause and CVD deaths [2]. Although men appear to be at higher absolute risk, hazard ratio curves associating all-cause and CVD mortality risk with reduced kidney function (lower eGFR range) are steeper in women, indicating accelerated risk relative to their male counterparts [4].

However, the relationship of gender with adverse outcomes—including CVD and death—appears to be more complex in people with diabetes. Several studies show that compared to men, women with diabetes may be at increased risk of CVD (incident coronary heart disease [CHD], heart failure), all-cause death and CVD death [5, 6]. Studies also show that younger age confers an independent risk of all-cause death and CVD death in diabetic individuals compared to those without diabetes, and in particular in those with greater severity of kidney disease [7]. However, the relationship among kidney function, gender and age on all-cause and CVD mortality in individuals with diabetes remains unstudied.

Our main objective in the present analysis was to determine whether the association between eGFR on all-cause mortality is modified by gender and age in individuals with diabetes. The secondary objective was to assess the same association on the secondary outcome of CVD mortality.

Our working hypothesis was that age category would be a factor influencing risk relationships at the lower end of the eGFR range showing accelerated risk of all-cause and CVD mortality in younger diabetic women relative to their male counterparts.

## Methods

### Study population

Singapore is a multi-ethnic country with major ethnic groups of Chinese, Malays, and Indians. In 2017 there were 18 polyclinics (primary care clinics) located throughout Singapore where about 60% of Singaporeans with hypertension and diabetes sought care. The 18 polyclinics were managed by two major healthcare groups prior to 2017, the SingHealth group and the National Healthcare group. The current study was derived from the electronic health records (EHR) of the SingHealth Diabetes Registry with the follow-up duration of 6 years [8]. Individuals with T2D receive annual blood and urine tests for serum creatinine and urine albumin, respectively.

Eligibility criteria for our study were individuals with type 2 diabetes (T2D) who had serum creatinine and urine albumin (quantitative or dipstick) recorded in EHR from the SingHealth Diabetes Registry in 2013. Baseline eGFR was calculated by Chronic Kidney Disease Epidemiology Collaboration (CKD-EPI) equation based on serum creatinine levels [9]. This equation has been validated in the population in Singapore [10]. We excluded individuals having eGFR levels ≥120 mL/min/1.73 m^2^ as that could reflect spuriously low serum creatinine in frail or malnourished individuals, and also because hyperfiltration may increase the risk of mortality among individuals with T2D [2, 11, 12]. The SingHealth Centralized Institutional Review Board granted ethics approval and consent waiver.

### Outcome assessment

Deaths were determined via linkage with the population-based Singapore Registry of Births and Deaths. Linkage was accomplished by matching the National Registration Identity Card number assigned to each citizen or permanent resident in Singapore and then verified by name. CVD mortality was identified as death from the following International Classification of Disease (ICD) codes for primary cause of death: ICD-10 codes I00–125, 150, 160–169, 170–171 for disease of circulatory system and R96 for sudden death.

### Exposure assessment and study covariates

The primary exposure variable was eGFR calculated from CKD-EPI equation based on serum creatinine [9]. All available eGFR values were averaged for 2013 for each patient. Information on covariates was also obtained from EHR data. Albuminuria was categorised based on semi-quantitative measurement of microalbumin based on urinary dipstick with values 20 mg/L or higher categorized as abnormal [13]. Other covariates included age, gender, socioeconomic status, smoking, and clinical information. Socioeconomic status was represented by living in rental housing (yes, no). Rental housing is a proxy indicator of low socioeconomic status in Singapore as shown in some studies [14]. Smoking was dichotomised as yes (for current smokers) and no (for former and never smokers). Comorbidities included hypertension (yes, no) and history of cardiovascular disease (yes, no). Other clinical information included intakes of lipid and anti-hypertensive medication (yes, no), systolic blood pressure (mmHg), diastolic blood pressure (mmHg), as well as levels of high-density lipoprotein (HDL) cholesterol (mmol/L), low-density lipoprotein (LDL) cholesterol (mmol/L), and triglycerides (mmol/L). Body mass index (BMI) was calculated as weight (kg) divided by height (m) squared. Creatinine measurements were calibrated to be traceable to isotope dilution mass spectrometry (IDMS) standardization [15, 16].

### Statistical analysis

Comparison of baseline characteristics was performed between men and women, stratified by age category (< 65 years, ≥65 years), using a chi-square test for categorical variables and an ANOVA F-test test for continuous variables. The effect size of standardized mean differences was estimated by Cramer’s V for counts and Cohen’s d for means. For Cramer’s V, absolute values of < 0.3, 0.3- < 0.5, and ≥ 0.5 represent small, medium, and large effect sizes, respectively [17]. For Cohen’s d, absolute values of < 0.5, 0.5- < 0.8, and ≥ 0.8 represent small, medium, and large effect sizes, respectively [17].

The Cox proportional hazards model was used to estimate hazard ratios for eGFR measurements in 2013 associated with risks of all-cause mortality and CVD mortality during 2013 to 2019 after adjustment for study covariates: age, gender, ethnicity (Chinese, Malays, Indians, and others), living in rental housing, smoking, BMI, using lipid lowering medications, albuminuria, systolic blood pressure (BP), diastolic BP, history of hypertension, history of CVD, levels of HDL cholesterol, LDL cholesterol and triglycerides. The covariates included in the model were statistically significant based on univariate analysis for at least one of the age group (< 65 years vs ≥ 65 years) when comparing between male and female. The model complies with the proportional hazards assumption.

For continuous eGFR, restricted cubic spline analysis with four knots (5th, 25th, 75th, and 95th percentile) and their product with gender were fitted for all-cause mortality and CVD mortality. Gender-specific reference points were used (eGFR of 90 mL/min per 1.73 m^2^), and from this model, the interaction was evaluated as the ratio of hazard ratios in women versus men at each 1 mL/min per 1.73 m^2^ of eGFR from 30 to 120 mL/min per 1.73 m^2^. To visually assess the main effect of gender on estimates of risk, analyses were repeated using a single eGFR reference point of 90 mL/min/1.73m^2^ in women. The two-way (eGFR×gender) and three-way interaction among eGFR, gender, and age (< 65 vs ≥65 years) as product of eGFR×gender×age for the outcome of all-cause mortality and CVD mortality were assessed in the restricted cubic spline analysis. Statistical significance was set at two-sided *P* < 0.05.

To evaluate the robustness of our results, we also included (i) a parsimonious model, (ii) a simplified model and (iii) a simplified model (excluding living in rental block). The parsimonious model adjusted for age, gender and albuminuria; the simplified model adjusted for age, gender, ethnicity, living in rental block, smoking, body mass index, lipid medications, hypertension medications, established CVD and albuminuria; the simplified model (excluding living in rental block) adjusted for all the covariates in the simplified model but excluded the variable on ‘living in rental block’. We conducted several sensitivity analyses including evaluations of: 1) Kaplan-Meier curves for the survival probability of men and women for mortality stratified by eGFR categories in the full model (Supplemental Fig. S1); 2) two-way interaction between eGFR×gender in the parsimonious model (Supplemental Fig. S2); 3) three-way interaction between eGFR×gender×age using a restricted cubic spline analysis on continuous eGFR with knots at 30, 45, 90 and 105 mL/min/1.73m^2^ in the full model (Supplemental Fig. S3); 4) three-way interaction between eGFR×gender×age in dataset including the average of at least two measurments of eGFR for every patient during 2013 to 2019 (*N* = 32,873) as the predictor variable in the full model (Supplemental Fig. S4); 5) three-way interaction between eGFR×gender×age primary analysis accounting for quantitative urine albumin creatinine ratio based on first available measurement between 2013 to 2019 (*N* = 32,087) in the parsimonious model (Supplemental Fig. S5); 6) two-way interaction between eGFR×gender in the parsimonious model for CVD mortality outcome (Supplemental Fig. S6); 7) three-way interaction between eGFR×gender×age in the full model for CVD mortality (Supplemental Fig. S7); 8) three-way interaction between eGFR×gender×age in the simplified model (Supplemental Fig. S8); 9) three-way interaction between eGFR×gender×age in the simplified model (excluding living in rental block) (Supplemental Fig. S9); 10) three-way interaction between eGFR×gender×age in the simplified model for CVD mortality (Supplemental Fig. S10); 11) three-way interaction between eGFR×gender×age in the simplified model (excluding living in rental block) for CVD mortality (Supplemental Fig. S11).

All the models in the abovementioned sensitivity analyses are for all-cause mortality outcome unless stated otherwise.

We also modelled eGFR as a categorical variable and compared the risks of all-cause mortality and CVD mortality among four categories of eGFR (< 30, 30- < 60, 60- < 90, and 90–120 mL/min per 1.73 m^2^) stratified by age and gender. Three-way interaction among eGFR, gender, and age (< 65 vs ≥65 years) as product of eGFR×gender×age for the outcome of all-cause mortality and CVD mortality were assessed in the analysis using categorical eGFR variable. In the unadjusted analysis (Table 2) and the simplified model (excluding living in rental block) (Table 3), eGFR between 90 and 119 mL/min per 1.73 m^2^ was used as reference in men and women. Rental block is excluded in Table 3 as its effect size in low. The sensitivity analysis using the categorical eGFR variable is shown in the simplified model (Supplemental Table S1).

For statistical significance of two-way and three-way interaction terms, a likelihood ratio test was used to compare a model with the interaction term and a nested model without the interaction term [18]. R studio (version 4.1.1) was used as the statistical software for all data analyses.

## Results

**Fig. 1:**
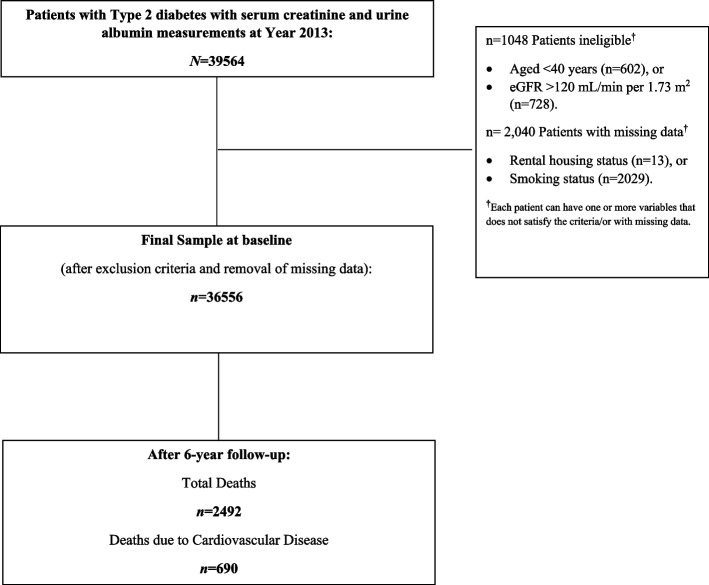
Participant selection flowchart

At baseline, 39,564 individuals were identified for eligibility screening. The final sample of 36,556 (92.3%) individuals with T2D was included in the present analysis. The flowchart detailing the study design is shown in Fig. 1**.** During mean follow-up of 6 years, 2492 (6.8%) individuals died from all causes, of which 690 (27.7%) deaths were due to CVD.

Table 1 compare the baseline characteristics of individuals with T2D stratified by gender and age category (< 65 years, ≥65 years). Among the 36,556 participants, mean age was 64.7 (SD: 10.0) years, 47.4% were male, 74.1% were Chinese, 12.7% were Malays, and 9.5% were Indians. Compared to men, women tended to smoke less and have higher HDL cholesterol in both age groups with significantly moderate to large effect sizes (Cohen’s d: < 65 years, 0.72; ≥65 years, 0.61). Younger women had lower diastolic BP compared to younger men, 69.3 (7.0) vs. 73.5 (6.7) mm Hg, respectively, (Cohen’s d, 0.61). No other covariates exhibited clinically important differences between men and women.

**Table 1:** Baseline characteristics by age and gender

Characteristics	Overall *N* = 36,556	< 65 years		≥65 years	
		Male *n* = 9376	Female *n* = 8974	Male *n* = 7966	Female *n* = 10,240
Age (years), Mean (SD)	64.7 (10.0)	56.2 (5.9)	57.0 (5.5)	72.3 (5.9)	73.2 (6.1)
Ethnicity, n (%)					
Chinese	27,185 (74.1)	6790 (72.4)	5733 (63.9)	6466 (81.2)	8196 (80.0)
Malay	4640 (12.7)	1188 (12.7)	1779 (19.8)	673 (8.5)	1000 (9.8)
Indian	3464 (9.5)	1032 (11.0)	1113 (12.4)	586 (7.4)	733 (7.2)
Others	1267 (3.5)	366 (3.9)	349 (3.9)	241 (3.0)	311 (3.0)
Rental housing, n (%)	2183 (6.0)	447 (4.8)	548 (6.1)	501 (6.3)	687 (6.7)
Smoking, n (%)	2873 (7.9)	1653 (17.6)	135 (1.5)	943 (11.8)	142 (1.4)
Body mass index (kg/m^2^), Mean (SD)^d^	26.2 (4.0)	26.7 (3.8)	27.1 (4.6)	25.2 (3.4)	25.6 (4.0)
Lipid medications, n (%)	31,409 (85.9)	7826 (83.5)	7578 (84.4)	6923 (86.9)	9082 (88.7)
Hypertension, n (%)	34,987 (95.7)	8763 (93.5)	8306 (92.6)	7819 (98.2)	10,099 (98.6)
History of cardiovascular disease, n (%)	6755 (18.5)	1607 (17.1)	682 (7.6)	2461 (30.9)	2005 (19.6)
Albuminuria, n (%)					
Abnormal	20,971 (57.4)	4491 (47.9)	4726 (52.7)	4840 (60.8)	6914 (67.5)
eGFR (mL/min per 1.73 m^2^), Mean (SD)	84.6 (19.5)	91.3 (15.5)	96.6 (15.5)	73.7 (17.8)	76.5 (19.0)
eGFR (mL/min per 1.73 m^2^), n (%)					
< 30	268 (0.7)	11 (0.1)	15 (0.2)	92 (1.2)	150 (1.5)
30 - < 60	4452 (12.2)	402 (4.3)	330 (3.7)	1745 (21.9)	1975 (19.3)
60 - < 90	14,562 (39.8)	3128 (33.4)	1722 (19.2)	4581 (57.5)	5131 (50.1)
90 - < 120	17,274 (47.3)	5835 (62.2)	6907 (77)	1548 (19.4)	2984 (29.1)
SBP (mmHg), Mean (SD)^e^	130.1 (11.5)	128.6 (11.0)	128.7 (11.7)	130.4 (11.5)	132.6 (11.4)
DBP (mmHg), Mean (SD)^f^	69.1 (7.2)	73.5 (6.7)	69.3 (7.0)	67.7 (6.7)	66.0 (6.3)
HDL cholesterol (mmol/L), Mean (SD)^g^	1.37 (0.4)	1.22 (0.3)	1.44 (0.3)	1.30 (0.3)	1.51 (0.4)
LDL cholesterol (mmol/L), Mean (SD)^h^	2.31 (0.7)	2.41 (0.7)	2.40 (0.7)	2.18 (0.6)	2.23 (0.6)
Triglycerides (mmol/L), Mean (SD)^i^	1.42 (0.7)	1.52 (0.8)	1.43 (0.7)	1.33 (0.6)	1.41 (0.6)

Abbreviations: *CVD* cardiovascular disease, *SBP* Systolic blood pressure, *DBP* Diastolic blood pressure, *HDL* high-density lipoprotein, *LDL* low-density lipoprotein

^a^Data are count (percentage) and mean (SD). Note: Missing values imputed in mean (SD) calculation except for body mass index

^d^5025 (13.7%) missing values were imputed with the national cutoff of body mass index between moderate and high risk of weight-related health problems [3] (27.5 kg/m^2^)

^e^716 (1.96%) missing values were imputed with the mean value of systolic blood pressure (132.3 mmHg)

^f^719 (1.97%) missing values were imputed with the mean value of diastolic blood pressure (69.5 mmHg)

^g^152 (0.42%) missing values were imputed with the mean value of HDL cholesterol (1.314 mmol/L)

^h^205 (0.56%) missing values were imputed with the mean value of LDL cholesterol (2.393 mmol/L)

^i^87 (0.24%) missing values were imputed with the mean value of triglycerides (1.541 mmol/L)

**Fig. 2:**
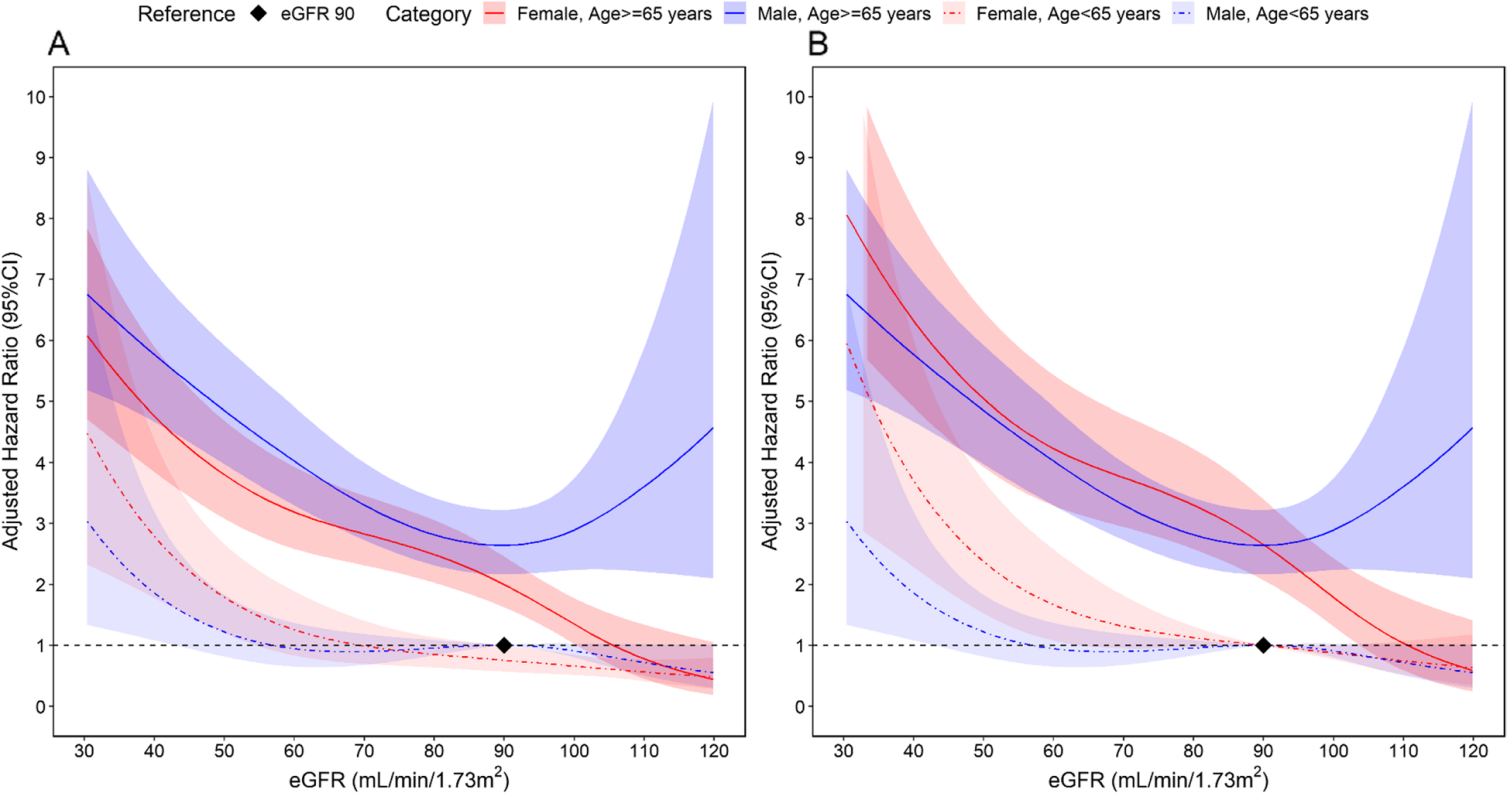
Cox regression model all-cause mortality hazard curves adjusted for covariates characterizing 3-way interactions of eGFR, gender and age category (*N* = 36,556; years 2013–2019). **A** Reference is eGFR 90 mL/min/1.73m^2^ in men aged < 65 years. **B** Reference for men and women is eGFR 90 mL/min/1.73m^2^ aged < 65 years. Model: Elapsed time to all-cause mortality = eGFR + age + gender + eGFR*age + eGFR*gender + age*gender + eGFR*age*gender + covariates. Covariates: ethnicity, living in rental block, smoking, body mass index, lipid medications, hypertension, established CVD, albuminuria, systolic blood pressure, diastolic blood pressure, high-density lipoprotein, low-density lipoprotein, and triglycerides. Restrictive cubic spline was applied to eGFR with knots at 5th, 25th, 75th and 95th percentile. Abbreviations: eGFR, estimated glomerular filtration rate. CVD, cardiovascular disease

We observed a significant three-way interaction among age, gender and eGFR relative to risk of all-cause mortality (*p* = 0.021) (Fig. 2). As shown in Fig. 2 panel A, using eGFR of 90 mL/min/1.73m^2^ as the reference point in younger men (aged < 65 years), younger women (aged < 65 years) had progressively higher risk of mortality at eGFR levels less than 70 ml/min/1.73m^2^. However, both older men and women (aged 65 years or older) had significantly higher risks of all-cause mortality at eGFR less than 90 mL/min/1.73m^2^ (Fig. 2 panel A).

As shown in Fig. 2 panel B, using eGFR of 90 mL/min/1.73m^2^ as the reference point in both younger (aged < 65 years) men and women, the hazard curves for all-casue mortality demonstrated the lowest risks at the highest eGFR levels and showed a steeper rise with decreasing eGFR in younger women (aged < 65 years) compared to younger (aged < 65 years) men. Among older (aged 65 years or older) women and men, the risks of all-cause mortality were similar at eGFR < 90 mL/min/1.73m^2^ (Fig. 2 panel B). Using age- and gender-specific eGFR of 90 ml/min/1.73m^2^ as the reference point, the adjusted hazard rate (HR) (95% CI) for all-cause mortality at eGFR 40 was 3.70 (2.29 to 5.99) in younger women and 1.86 (1.08 to 3.19) in younger men. The corresponding adjusted HR were 2.38 (2.02 to 2.82) and 2.18 (1.85 to 2.57) in older women and older men, respectively.

The survival probabilities were lowest for older (> 65 years) men and younger (< 65 years) women with eGFR < 30 ml/min/1.73m^2^ (Supplemental Fig. S1). These results remained consistent in all sensitivity analyses of all-cause mortality with persistent significant interaction *p*-values (ranging from *p* = 0.03 to *p* < 0.001) in analyses of two-way interactions (eGFR×gender) and three-way interactions under various stratifications and use of differing eGFR knots in 1) a model using subjects with at least two measurments of eGFR (*N* = 32,873) and 2) a model replacing presence of dipstick albuminuria by quantitative urine albumin-to-creatinine ratio (*N* = 32,087) (Supplemental Figs. S2 to S5, S8 and S9).

When analyzing eGFR as a categorical variable, we observed consistent results for all-cause mortality (Table 2). For example, when using eGFR of 90 to 120 mL/min/1.73m^2^ as the reference in younger (aged < 65 years) women and men, younger women with eGFR < 60 to 30 mL/min/1.73m^2^ exhibited a significantly higher mortality risk (HR, 3.01 [95% confidence interval: 1.84, 4.94]) compared to their male counterparts (HR, 1.44 [0.88, 2.38]) (Table 2). However, the mortality risks were comparable in older (65 years or older) women and men with eGFR < 60 to 30 mL/min/1.73m^2^ (HRs, 3.03 [2.47, 3.71] and 2.05 [1.69, 2.47], respectively) (Table 2). These results remained consistent when for absolute mortality rates (expressed incidence per as 1000 person years) (Table 2) and for mortality risk inthe simplified model (excluding living in rental block) (Table 3) and the simplified model (Supplemental Table S1).

**Table 2:** Cox regression model eGFR hazard ratios for all-cause and CVD mortality among patients with type 2 diabetes stratified by age using 65 years as cut-off and gender, with eGFR between 90 and 119 mL/min per 1.73 m^2^ as reference (*N* = 36,556; years 2013–2019)

eGFR categories (mL/min per 1.73 m^2^)	< 65 years				≥65 years			
	Number of Deaths (incidence rate (%) Male^a^)	Hazard Ratio (95% CI) Male	Number of Deaths (incidence rate (%) Female^a^)	Hazard Ratio (95% CI) Female	Number of Deaths (incidence rate (%) Male^a^)	Hazard Ratio (95% CI) Male	Number of Deaths (incidence rate (%) Female^a^)	Hazard Ratio (95% CI) Female
**All-cause mortality**								
< 30	2 (31.25)	6.63 (1.64, 26.70)	5 (64.94)	22.88 (9.36, 55.94)	32 (66.12)	4.19 (2.86, 6.13)	42 (51.92)	6.90 (4.89, 9.74)
30 - < 60	17 (7.13)	1.44 (0.88, 2.38)	18 (9.23)	3.01 (1.84, 4.94)	333 (33.89)	2.05 (1.69, 2.47)	270 (23.65)	3.03 (2.47, 3.71)
60 - < 90	103 (5.54)	1.12 (0.88, 1.43)	53 (5.17)	1.68 (1.22, 2.32)	536 (20.19)	1.20 (1.00, 1.44)	489 (16.30)	2.06 (1.71, 2.49)
90 - < 120	172 (4.95)	1.00 (Reference)	127 (3.08)	1.00 (Reference)	152 (16.90)	1.00 (Reference)	141 (7.99)	1.00 (Reference)
**CVD mortality**								
< 30	1 (15.63)	9.58 (1.33, 69.14)	3 (38.96)	59.64 (18.16, 195.91)	9 (18.60)	4.72 (2.28, 9.79)	17 (21.01)	14.43 (7.87, 26.48)
30 - < 60	8 (3.35)	1.98 (0.95, 4.15)	7 (3.59)	5.13 (2.25, 11.71)	95 (9.67)	2.38 (1.63, 3.48)	88 (7.71)	5.13 (3.33, 7.89)
60 - < 90	37 (1.99)	1.17 (0.78, 1.77)	12 (1.17)	1.67 (0.85, 3.27)	143 (5.39)	1.31 (0.91, 1.88)	118 (3.93)	2.59 (1.71, 3.94)
90 - < 120	59 (1.70)	1.00 (Reference)	29 (0.70)	1.00 (Reference)	37 (4.11)	1.00 (Reference)	27 (1.53)	1.00 (Reference)

Unadjusted model: *Abbreviations*: *eGFR* estimated glomerular filtration rate, *CVD* cardiovascular disease

^a^The incidence rate represented is for per 1000 person-years

**Table 3:** Cox regression simplified model (excluding living in rental block) eGFR hazard ratios for all-cause and CVD mortality among patients with type 2 diabetes stratified by age using 65 years as cut-off and gender, with eGFR between 90 and 119 mL/min per 1.73 m^2^ as reference (N = 36,556; years 2013–2019)

eGFR categories (mL/min per 1.73 m^2^)	< 65 years		≥65 years	
	Male	Female	Male	Female
**All-cause mortality**				
< 30	4.97 (1.23, 20.04)	18.22 (7.49, 44.32)	3.26 (2.22, 4.78)	5.73 (4.05, 8.10)
30 - < 60	1.29 (0.79, 2.13)	2.75 (1.68, 4.51)	1.88 (1.55, 2.28)	2.63 (2.14, 3.22)
60 - < 90	1.12 (0.88, 1.43)	1.64 (1.19, 2.25)	1.17 (0.98, 1.40)	1.92 (1.59, 2.32)
90 - < 120	1.00 (Reference)	1.00 (Reference)	1.00 (Reference)	1.00 (Reference)
**CVD mortality**				
< 30	6.11 (0.84, 44.20)	40.63 (13.09, 126.15)	2.80 (1.35, 5.84)	10.02 (5.44, 18.46)
30 - < 60	1.51 (0.72, 3.16)	4.03 (1.76, 9.22)	1.87 (1.27, 2.74)	3.87 (2.51, 5.97)
60 - < 90	1.09 (0.73, 1.65)	1.50 (0.77, 2.95)	1.19 (0.83, 1.72)	2.22 (1.46, 3.37)
90 - < 120	1.00 (Reference)	1.00 (Reference)	1.00 (Reference)	1.00 (Reference)

Model: Elapsed time to all-cause/CVD mortality = eGFR + age + gender + eGFR*age + eGFR*gender + age*gender + eGFR*age*gender + covariates. Covariates: ethnicity, smoking, body mass index, lipid medications, established CVD, albuminuria. Abbreviations: *eGFR* estimated glomerular filtration rate, *CVD* cardiovascular disease

Similar to all-cause mortality, the association between eGFR and CVD mortality was more pronounced for women with higher risks at the lowest eGFR levels and generally steeper declines in risk with increasing eGFR levels. The gender×eGFR interaction was statistically significant (*p* < 0.001) (Supplemental Fig. S6). However, the three-way interaction of age×gender×eGFR for CVD mortality did not achieve statistical significance in the main analysis (Supplemental Figs. S7, S10 and S11).

## Discussion

Reduced kidney function measured using eGFR is associated with an increase in the risk of all-cause mortality and CVD mortality in patients with and without diabetes [3]. In our analysis of 36,556 patients with T2D in multi-institutional diabetes registry in Singapore followed for up to 6 years, we observed a statistically significant 3-way interaction among eGFR, gender and age indicating that younger women with lower eGFR are at 2 to 6 times higher risk of dying from any cause than the younger men counterparts. Our analysis accounted for several conventional CVD risk factors including BMI, BP, fasting glucose, cholesterol levels, anti-hypertensive and lipid lowering medications, and proteinuria. No significant differences in mortality were observed in elderly women versus elderly men at low eGFR values. Although the 3-way interaction test was not statistically significant for the outcome of CVD mortality—possibly due to the sample size, a similar pattern of relationships among gender, eGFR and age was observed. Our findings underscore the urgency of risk stratification in younger women with diabetes and low GFR for targeted efforts to prevent all deaths—including CVD deaths.

Several studies and meta-analyses have shown linear increases in all-cause mortality and CVD mortality with declines in eGFR beyond a threshold of about 75 ml/min/1.73m^2^ [2, 3]. Although men appear to be at higher absolute risk, increases in risk of all-cause and CVD mortality as a function of declining eGFR occur at demonstrably accelerated rates in women relative to men [4]. Systematic reviews of studies in patients with diabetes reveal that women are at substantially increased risk of death, CVD death and incident CVD (incident CHD, heart failure) compared with men, although there was significant heterogeneity in cause of death among studies [6, 19]. Of note, women with diabetes have been shown to have up to 40% greater risk of incident CHD compared to diabetic men, in part due to an excess of CVD risk factors [5]. Other studies have shown that patients with diabetes, compared to those without diabetes, were at higher risk of death from any cause, and that risk of cardiovascular death in patients with diabetes, compared to those without diabetes, begins to rise at a younger age, and in particular in those with greater severity of kidney disease [7].

CVD mortality rates continue to rise in younger women in many countries and is often neglected [20, 21]. The associations among gender, kidney function and age with all-cause and CVD mortality have not previously been reported in patients with diabetes per se. Our findings are novel in elucidating the relationship between reduced kidney function and gender on excess mortality risk as modified by age: at lower eGFR, younger women compared to younger men have a significantly higher risk of death, while we found no significant difference in death risk between elderly women and elderly men.

The reasons for age-related effect modifications of the gender and eGFR relationship with mortality can potentially be attributed to both biological factors as well as those related to access to the health systems. First, diabetes has been found to negate the protective effect of the female sex against coronary heart disease and death from cardiovascular disease [22]. Although our analysis accounted for several conventional CVD risk factors, data on waist circumference or other measures of visceral adiposity, a known risk factor for cardiometabolic multimorbidity especially in Asians, were not available [23]. Second, low eGFR is associated with a host of “kidney specific” factors which include increased activity of renin-angiotensin system, neurohormonal activation, water and sodium retention, inflammation, vitamin D deficiency, calcium and phosphorus dysregulation, and increased asymmetric dimethyl arginine (ADMA) with greater bioavailability of nitric oxide, leading to endothelial dysfunction [24]. Studies show that endothelial dysfunction may develop at an earlier age in women relative to men who develop diabetes [25]. Third, women with diabetes and low eGFR are also likely to have history of inflammatory disorders such as rheumatoid arthritis, psoriasis, or lupus that are known to have robust associations with vascular disease and mortality that occur preferentially at a younger age [26–28]. Fourth, and perhaps most concerning, is the possibility of gender-related bias in treatment of young women with diabetes and reduced kidney function. Unfortunately, treatment bias against women has been reported for several conditions globally, including in the United States. For example, the analysis of the Atherosclerosis Risk in Communities (ARIC) Surveillance study conducted in 4 communities showed that young women with acute myocardial infarction were less likely to receive guideline-based therapies [29]. Of note, only a minority of patients with CKD who present with acute myocardial infarction (AMI) report the typical symptoms of chest, arm, shoulder, or neck pain [29, 30]. Our findings underscore the urgency to increase awareness of healthcare providers to high risk of death in young women with diabetes and CKD.

Lifestyle plus pharmacotherapy targeting blood pressure lowering preferably with Renin-angiotensin-aldosterone system inhibitor, lipid lowering, and antidiabetic agents including Sodium-glucose Cotransporter-2 inhibitors, glucagon-like peptide 1 agonists, as well as non-steroidal mineralocorticoids optimize CVD and kidney risk reduction in patients with diabetes and CKD [29, 31]. Our findings have tremendous implications for clinical practice and policy, and underscore the need for risk stratification of younger women with diabetes and lower eGFR and prompt institution of targeted strategies to prevent deaths. Awareness and prompt recognition of acute events is important for timely interventions for revascularization and secondary prevention [30].

Our study has several limitations. First, inherent to analysis of clinic-based registry and EHR is the possibility of an informed-presence bias as individuals who are ill (e.g. lower eGFR) are likely to seek healthcare, and get tested more frequently across a variety of settings (emergency room, outpatient clinic, hospital) compared to those who are not, with the greater possibility of detecting potentially spurious associations [32, 33]. However, we accounted for the presence of co-morbidities (hypertension, CVD), clinical factors (BP, lipids) and related medications. Therefore, we believe our findings are generalizable to the vast majority of patients with diabetes in Singapore. Second, information on education and physical activity was not available in the registry. These components of socioeconomic and lifestyle factors are associated with all-cause and CVD mortality [34]. However, our main findings of effect modification of the association between eGFR level and gender by age on mortality risk were consistent after accounting for BMI, housing type, smoking status, as well as clinical risk factors for CVD. Third, only semi-quantitative as opposed to quantitative measures of albuminuria were available for the vast majority of patients in 2013. The latter has a continuous, linear relationship with mortality and CVD deaths [2]. However, several studies have shown excellent validity of the semi-quantitative tests compared to quantitative methods [13, 35]. Moreover, sensitivity analysis accounting for ACR obtained during 2013 to 2019 yeilded consistent results. Fourth, the study findings might not be broadly applicable to other populations due to the specific healthcare settings within the diabetes group studied, potentially influencing the main results. Moreover, even though our study had a relatively sizable sample, there were fewer participants with low eGFR, notably in younger age strata. Furthermore, the number of deaths among younger women and men was limited. This scarcity influenced the precision of estimating mortality risks associated with low eGFR.

Additionally, our study lacked sufficient power to detect three-way interactions for specific causes of mortality, such as CVD deaths. Nevertheless, the risk estimates indicate a similar relationship and trends between CVD deaths and overall mortality, with younger women exhibiting a higher risk of CVD-related deaths compared to men with reduced eGFR levels.

Major strengths of our study include a large and well characterized cohort of patients with diabetes, follow-up over 6 years, linkage to the comprehensive national disease registry for death and cause of death records, and serum creatinine measurements being standardized to IDMS [9]. Moreover, sensitivity analysis using categorical cut-points and spline models with different knots for eGFR yielded consistent results. Thus, we believe our findings are robust.

## Conclusion

In conclusion, among patients with diabetes, we found a higher mortality of women compared to men confined to those with reduced kidney function and at younger age. No differences in mortality were observed in the elderly women vs men. Our results indicate that younger women with diabetes reduced kidney function are a very high-risk group deserving of special study and that patients’ age and kidney function must be considered in assessments of gender-based differences in mortality in patients with diabetes. Our results underscore the urgency of creating awareness among the providers and patients of gender-related disparities observed at low eGFR in patients with T2D.

### Supplementary Information

**Additional file 1: Supplementary Table S1.** Cox regression simplified model eGFR hazard ratios for all-cause and CVD mortality among patients with type 2 diabetes stratified by age using 65 years as cut-off and gender, with eGFR between 90-119 mL/min per 1.73 m2 as reference. **Supplemental Figure S1.** Kaplan-Meier curves for the survival probability of female and male among groups stratified by eGFR with all-cause mortality as outcome. **Supplemental Figure S2.** Cox regression parsimonious model all-cause mortality hazard curves adjusted for covariates characterizing 2-way interaction of eGFR and gender. **Supplemental Figure S3.** Cox regression sensitivity model with different eGFR knots for the outcome of all-cause mortality hazard curves adjusted for covariates characterizing 3-way interaction of eGFR, gender and age category. **Supplemental Figure S4.** Cox regression model all-cause mortality hazard curves adjusted for covariates characterizing 3-way interaction of eGFR, gender and age category in dataset including the average of at least two measurments of eGFR for every patient during 2013 to 2019. **Supplemental Figure S5.** Cox regression parsimonious model all-cause mortality hazard curves adjusted for covariates, replacing albuminuria by albumin-creatinine-ratio, characterizing 3-way interaction of eGFR, gender and age category. **Supplemental Figure S6.** Cox regression parsimonious model CVD mortality hazard curves adjusted for covariates characterizing 2-way interaction of eGFR and gender. **Supplemental Figure S7.** Cox regression model CVD mortality hazard curves adjusted for covariates characterizing 3-way interaction of eGFR, gender and age category. **Supplemental Figure S8.** Cox regression simplified model all-cause mortality hazard curves adjusted for covariates characterizing 3-way interactions of eGFR, gender and age category. **Supplemental Figure S9.** Cox regression simplified model (excluding living in rental block) all-cause mortality hazard curves adjusted for covariates characterizing 3-way interactions of eGFR, gender and age category. **Supplemental Figure S10.** Cox regression simplified model CVD mortality hazard curves adjusted for covariates characterizing 3-way interactions of eGFR, gender and age category. **Supplemental Figure S11.** Cox regression simplified model (excluding living in rental block) CVD mortality hazard curves adjusted for covariates characterizing 3-way interactions of eGFR, gender and age category.

## Data Availability

Data are available on reasonable request from the corresponding author subject to approval by the IRB.

## Abbreviations

ADMA: Asymmetric dimethyl arginine
AMI: Acute myocardial infarction
ARIC: Atherosclerosis Risk in Communities
BMI: Body mass index
BP: Blood pressure
CHD: Coronary heart disease
CI: Confidence interval
CKD-EPI: Chronic Kidney Disease Epidemiology Collaboration
CVD: Cardiovascular disease
eGFR: Estimated glomerular filtration rate
EHR: Electronic health records
ESKD: End-stage kidney disease
HDL: High-density lipoprotein
HR: Hazard rate
ICD: International Classification of Disease
IDMS: Isotope dilution mass spectrometry
LDL: Low-density lipoprotein
T2D: Type 2 diabetes
