# Supplementary material for: Heterogeneity by age and gender in the association of kidney function with mortality among patients with diabetes - analysis of diabetes registry in Singapore
Source: BMC Nephrol. 2024 Jan 17;25:23. doi: 10.1186/s12882-024-03463-8 (PMC10795380; doi:10.1186/s12882-024-03463-8)
Supplement: Supplementary file 1 — Additional file 1: Supplementary Table S1. Cox regression simplified model eGFR hazard ratios for all-cause and CVD mortality among patients with type 2 diabetes stratified by age using 65 years as cut-off and gender, with eGFR between 90-119 mL/min per 1.73 m2 as reference. Supplemental Figure S1. Kaplan-Meier curves for the survival probability of female and male among groups stratified by eGFR with all-cause mortality as outcome. Supplemental Figure S2. Cox regression parsimonious model all-cause mortality hazard curves adjusted for covariates characterizing 2-way interaction of eGFR and gender. Supplemental Figure S3. Cox regression sensitivity model with different eGFR knots for the outcome of all-cause mortality hazard curves adjusted for covariates characterizing 3-way interaction of eGFR, gender and age category. Supplemental Figure S4. Cox regression model all-cause mortality hazard curves adjusted for covariates characterizing 3-way interaction of eGFR, gender and age category in dataset including the average of at least two measurments of eGFR for every patient during 2013 to 2019. Supplemental Figure S5. Cox regression parsimonious model all-cause mortality hazard curves adjusted for covariates, replacing albuminuria by albumin-creatinine-ratio, characterizing 3-way interaction of eGFR, gender and age category. Supplemental Figure S6. Cox regression parsimonious model CVD mortality hazard curves adjusted for covariates characterizing 2-way interaction of eGFR and gender. Supplemental Figure S7. Cox regression model CVD mortality hazard curves adjusted for covariates characterizing 3-way interaction of eGFR, gender and age category. Supplemental Figure S8. Cox regression simplified model all-cause mortality hazard curves adjusted for covariates characterizing 3-way interactions of eGFR, gender and age category. Supplemental Figure S9. Cox regression simplified model (excluding living in rental block) all-cause mortality hazard curves adjusted for covariat [file 12882_2024_3463_MOESM1_ESM.docx]

**Supplementary Material**

**Table of Contents**

Supplemental Table S1**.** Cox regression simplified model eGFR hazard ratios for all-cause and CVD mortality among patients with type 2 diabetes stratified by age using 65 years as cut-off and gender, with eGFR between 90-119 mL/min per 1.73 m^2^ as reference8

Supplemental Figure S1. Kaplan-Meier curves for the survival probability of female and male among groups stratified by eGFR with all-cause mortality as outcome 10

Supplemental Figure S2. Cox regression parsimonious model all-cause mortality hazard curves adjusted for covariates characterizing 2-way interaction of eGFR and gender11

Supplemental Figure S3. Cox regression sensitivity model with different eGFR knots for the outcome of all-cause mortality hazard curves adjusted for covariates characterizing 3-way interaction of eGFR, gender and age category12

Supplemental Figure S4. Cox regression model all-cause mortality hazard curves adjusted for covariates characterizing 3-way interaction of eGFR, gender and age category in dataset including the average of at least two measurments of eGFR for every patient during 2013 to 2019 13

Supplemental Figure S5. Cox regression parsimonious model all-cause mortality hazard curves adjusted for covariates, replacing albuminuria by albumin-creatinine-ratio, characterizing 3-way interaction of eGFR, gender and age category14

Supplemental Figure S6. Cox regression parsimonious model CVD mortality hazard curves adjusted for covariates characterizing 2-way interaction of eGFR and gender15

Supplemental Figure S7. Cox regression model CVD mortality hazard curves adjusted for covariates characterizing 3-way interaction of eGFR, gender and age category16

Supplemental Figure S8. Cox regression simplified model all-cause mortality hazard curves adjusted for covariates characterizing 3-way interactions of eGFR, gender and age category17

Supplemental Figure S9**.** Cox regression simplified model (excluding living in rental block) all-cause mortality hazard curves adjusted for covariates characterizing 3-way interactions of eGFR, gender and age category18

Supplemental Figure S10. Cox regression simplified model CVD mortality hazard curves adjusted for covariates characterizing 3-way interactions of eGFR, gender and age category19

Supplemental Figure S11. Cox regression simplified model (excluding living in rental block) CVD mortality hazard curves adjusted for covariates characterizing 3-way interactions of eGFR, gender and age category20

|  |
| --- |

**Supplemental Table S1.** Cox regression simplified model eGFR hazard ratios for all-cause and CVD mortality among patients with type 2 diabetes stratified by age using 65 years as cut-off and gender, with eGFR between 90-119 mL/min per 1.73 m^2^ as reference (N=36556; years 2013-2019)

| **eGFR categories**  **(mL/min per 1.73 m^2^)** | **<65 years** | | | **≥65 years** | |
| --- | --- | --- | --- | --- | --- |
|  | **Male** | **Female** | | **Male** | **Female** |
| **All-cause mortality** |  | | |  | |
| <30 | 4.82 (1.19, 19.45) | 18.10 (7.44, 44.02) | | 3.30 (2.25, 4.85) | 5.67 (4.01, 8.02) |
| 30 - <60 | 1.30 (0.79, 2.14) | 2.73 (1.66, 4.47) | | 1.88 (1.55, 2.28) | 2.62 (2.13, 3.21) |
| 60 - <90 | 1.12 (0.88, 1.43) | 1.63 (1.18, 2.25) | | 1.18 (0.98, 1.41) | 1.92 (1.59, 2.31) |
| 90 - <120 | 1.00 (Reference) | 1.00 (Reference) | | 1.00 (Reference) | 1.00 (Reference) |
| **CVD mortality** |  | | | | |
| <30 | 5.95 (0.82, 43.03) | 40.27 (12.97, 125.03) | | 2.86 (1.38, 5.96) | 9.86 (5.36, 18.17) |
| 30 - <60 | 1.51 (0.72, 3.18) | 3.98 (1.74, 9.12) | | 1.86 (1.27, 2.73) | 3.86 (2.50, 5.96) |
| 60 - <90 | 1.10 (0.73, 1.66) | 1.49 (0.76, 2.93) | | 1.20 (0.83, 1.72) | 2.22 (1.46, 3.37) |
| 90 - <120 | 1.00 (Reference) | | 1.00 (Reference) | 1.00 (Reference) | 1.00 (Reference) |

Model: Elapsed time to all-cause/CVD mortality = eGFR + age + gender + eGFR*age + eGFR*gender + age*gender + eGFR*age*gender + covariates. Covariates: ethnicity, living in rental block, smoking, body mass index, lipid medications, established CVD, and albuminuria. **Abbreviations:** eGFR, estimated glomerular filtration rate. CVD, cardiovascular disease.

**Supplemental Figure 1.** Kaplan-Meier curves for the survival probability of female and male among groups stratified by eGFR with all-cause mortality as outcome (N=36556; years 2013-2019)


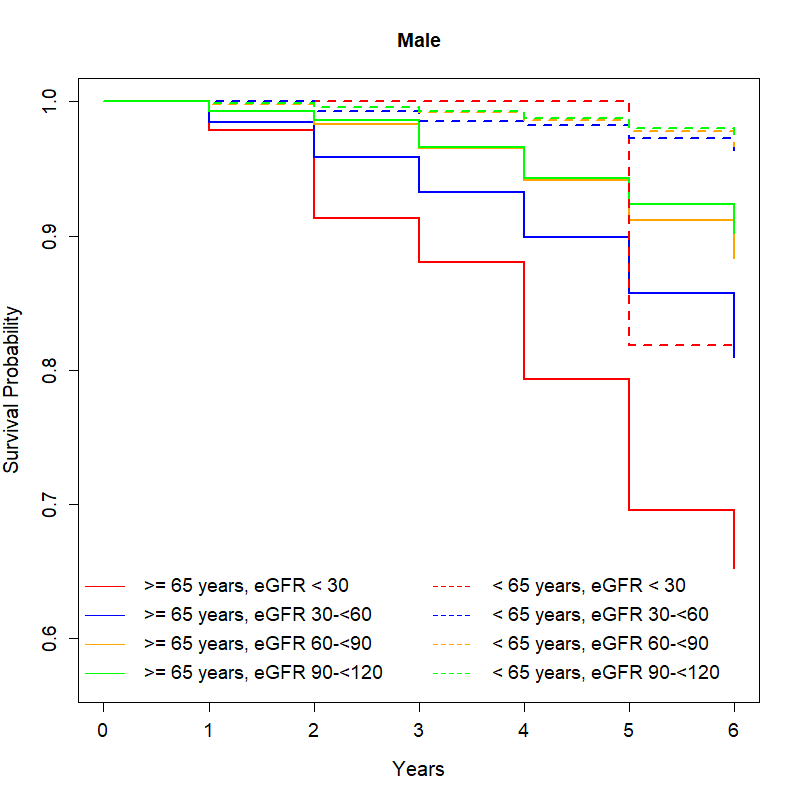

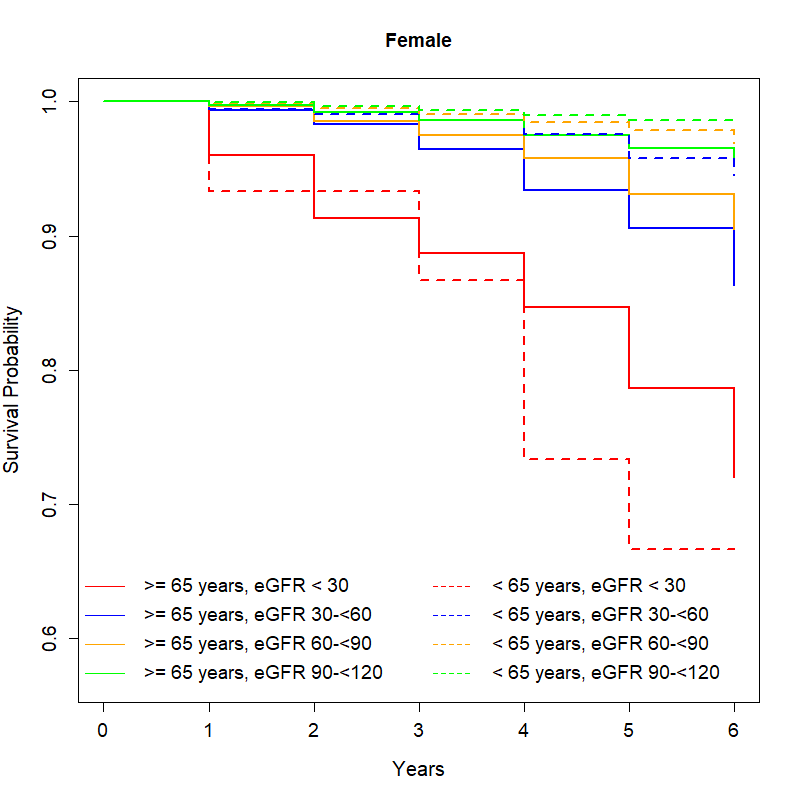


Note: The unit of GFR is in mL/min per 1.73 m^2^.

**Supplemental Figure S2.** Cox regression parsimonious model all-cause mortality hazard curves adjusted for covariates characterizing 2-way interaction of eGFR and gender (N=36556; years 2013-2019)


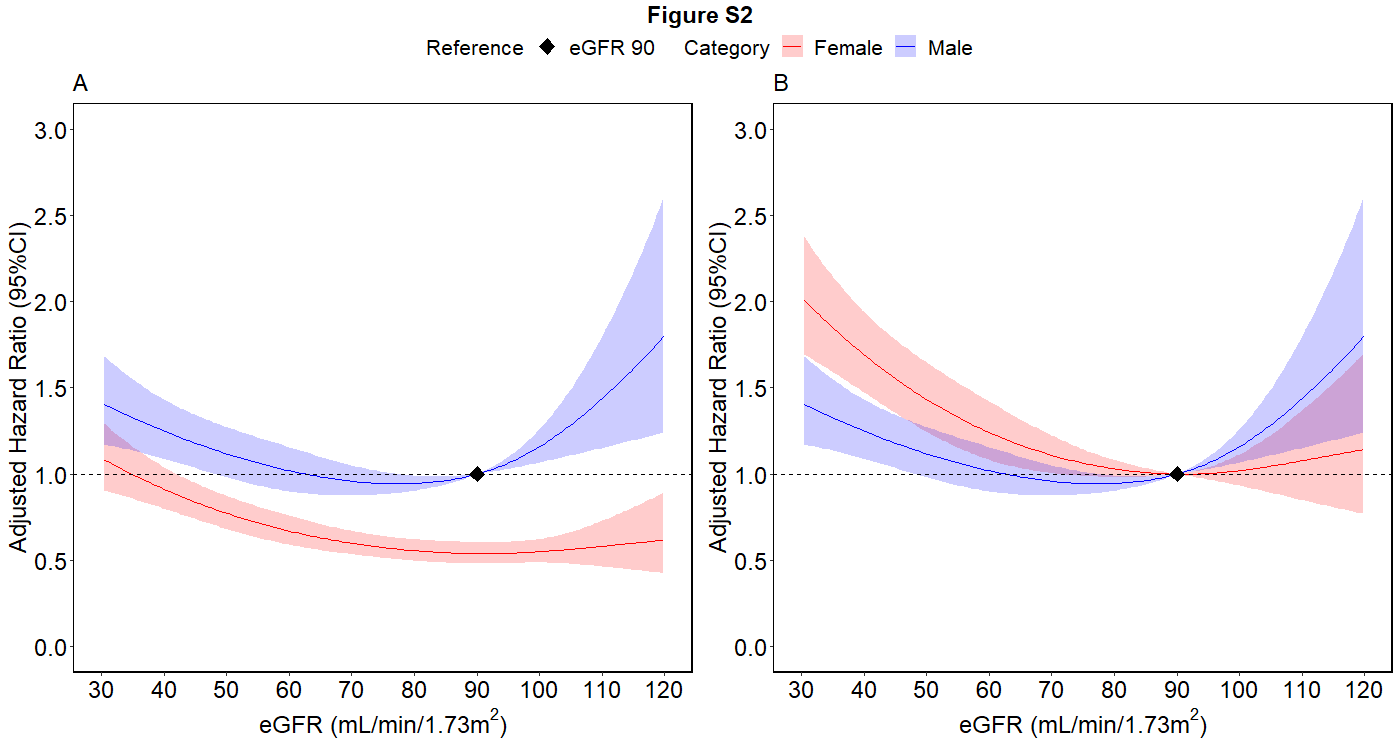


*eGFR × Gender interaction, p* < 0.001

**A** Reference is eGFR 90 mL/min/1.73m^2^ in men. **B** Reference for men and women is eGFR 90 mL/min/1.73m^2^. Model: Elapsed time to all-cause mortality = eGFR + gender + eGFR*gender + covariates. Covariates: age, albuminuria. Restrictive cubic spline was applied to eGFR with knots at 5^th^, 25^th^, 75^th^ and 95^th^ percentile. **Abbreviations:** eGFR, estimated glomerular filtration rate.

**Supplemental Figure S3.** Cox regression sensitivity model with different eGFR knots for the outcome of all-cause mortality hazard curves adjusted for covariates characterizing 3-way interaction of eGFR, gender and age category (N=36556; years 2013-2019)


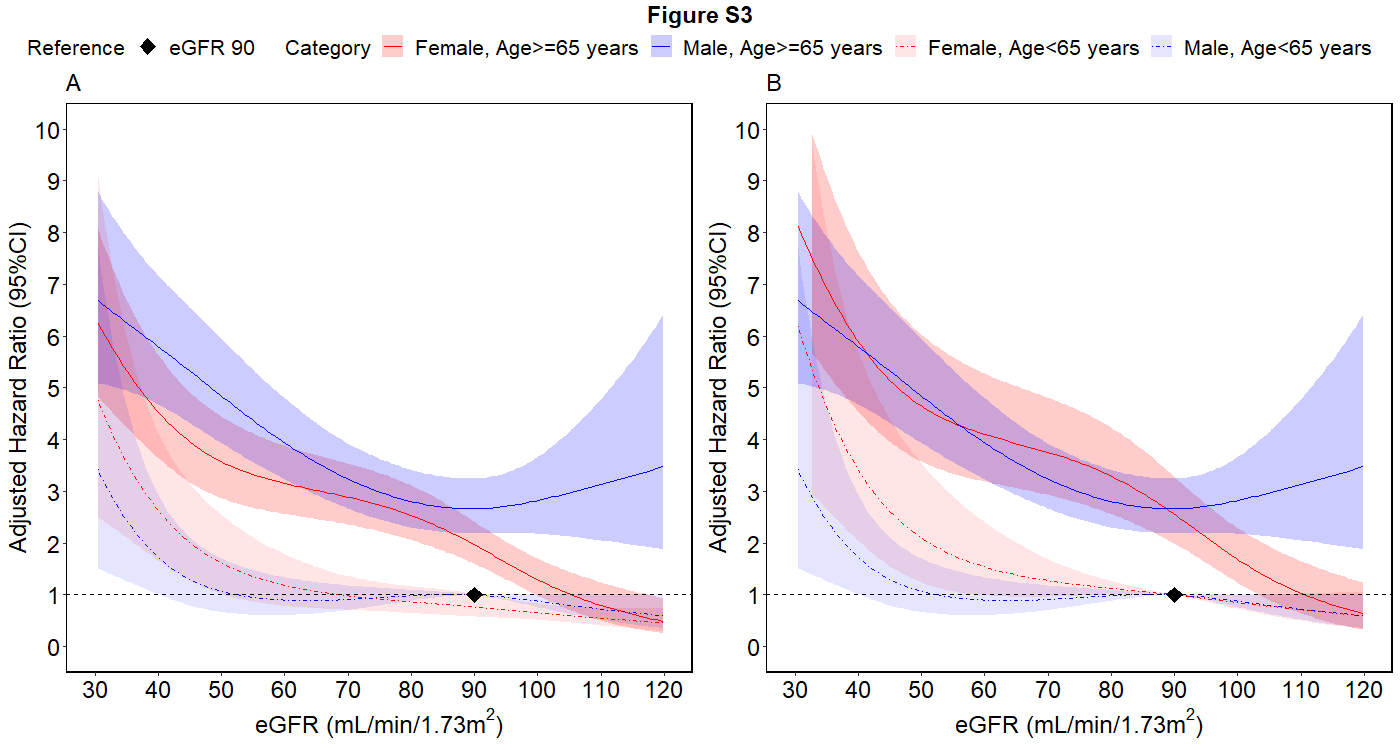


*eGFR × Age × Gender interaction, p* = 0.032

**A** Reference is eGFR 90 mL/min/1.73m^2^ in men aged <65 years. **B** Reference for men and women is eGFR 90 mL/min/1.73m^2^ aged <65 years. Model: Elapsed time to all-cause mortality = eGFR + age + gender + eGFR*age + eGFR*gender + age*gender + eGFR*age*gender + covariates. Covariates: ethnicity, living in rental block, smoking, body mass index, lipid medications, hypertension, established CVD, albuminuria, systolic blood pressure, diastolic blood pressure, high-density lipoprotein, low-density lipoprotein, and triglycerides. Restrictive cubic spline was applied to eGFR with knots at 30, 45, 90 and 105 mL/min per 1.73 m^2^. **Abbreviations:** eGFR, estimated glomerular filtration rate. CVD, cardiovascular disease.

**Supplemental Figure S4.** Cox regression model all-cause mortality hazard curves adjusted for covariates characterizing 3-way interaction of eGFR, gender and age category in dataset including the average of at least two measurments of eGFR for every patient during 2013 to 2019 (N=32873; years 2013-2019)


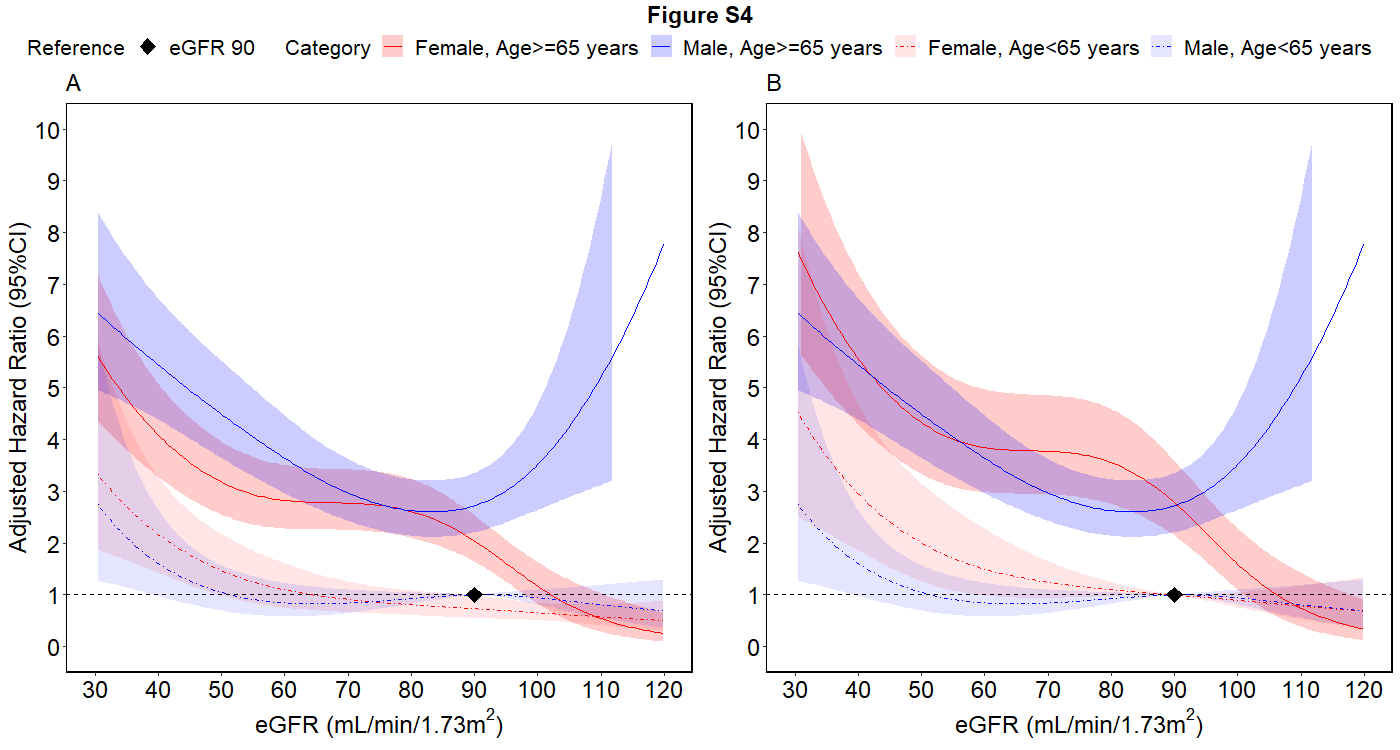


*eGFR × Age × Gender interaction, p* <0.001

**A** Reference is eGFR 90 mL/min/1.73m^2^ in men aged <65 years. **B** Reference for men and women is eGFR 90 mL/min/1.73m^2^ aged <65 years. Model: Elapsed time to all-cause mortality = eGFR + age + gender + eGFR*age + eGFR*gender + age*gender + eGFR*age*gender + covariates. Covariates: ethnicity, living in rental block, smoking, body mass index, lipid medications, hypertension, established CVD, albuminuria, systolic blood pressure, diastolic blood pressure, high-density lipoprotein, low-density lipoprotein, and triglycerides. Restrictive cubic spline was applied to eGFR with knots at 5^th^, 25^th^, 75^th^ and 95^th^ percentile. **Abbreviations:** eGFR, estimated glomerular filtration rate. CVD, cardiovascular disease.

**Supplemental Figure S5.** Cox regression parsimonious model all-cause mortality hazard curves adjusted for covariates, replacing albuminuria by albumin-creatinine-ratio, characterizing 3-way interaction of eGFR, gender and age category (N=32087; years 2013-2019)


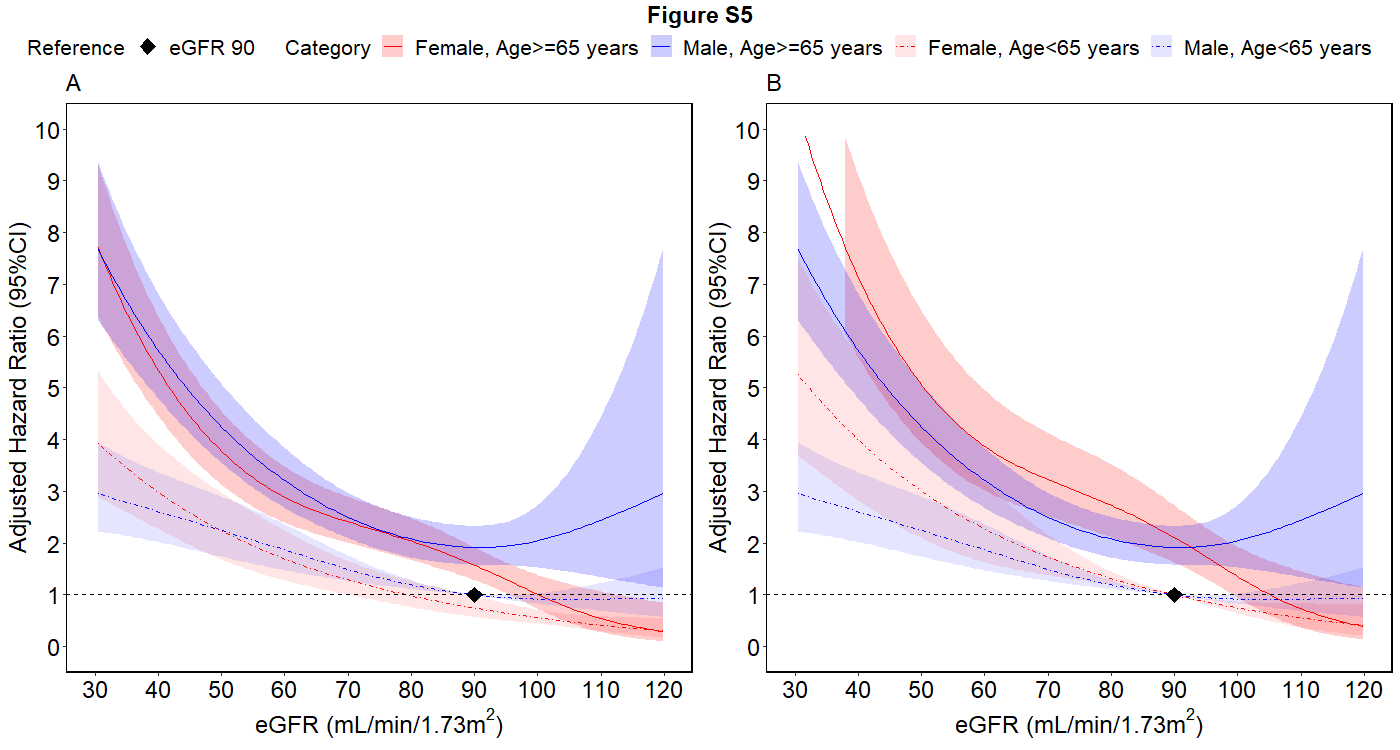


*eGFR × Age × Gender interaction, p* < 0.001

**A** Reference is eGFR 90 mL/min/1.73m^2^ in men aged <65 years. **B** Reference for men and women is eGFR 90 mL/min/1.73m^2^ aged <65 years. Model: Elapsed time to all-cause mortality = eGFR + gender + eGFR*gender + covariates. Covariates: age, albumin-to-creatinine ratio. Restrictive cubic spline was applied to eGFR with knots at 5^th^, 25^th^, 75^th^ and 95^th^ percentile. **Abbreviations:** eGFR, estimated glomerular filtration rate.

**Supplemental Figure S6.** Cox regression parsimonious model CVD mortality hazard curves adjusted for covariates characterizing 2-way interaction of eGFR and gender (N=36556; years 2013-2019)


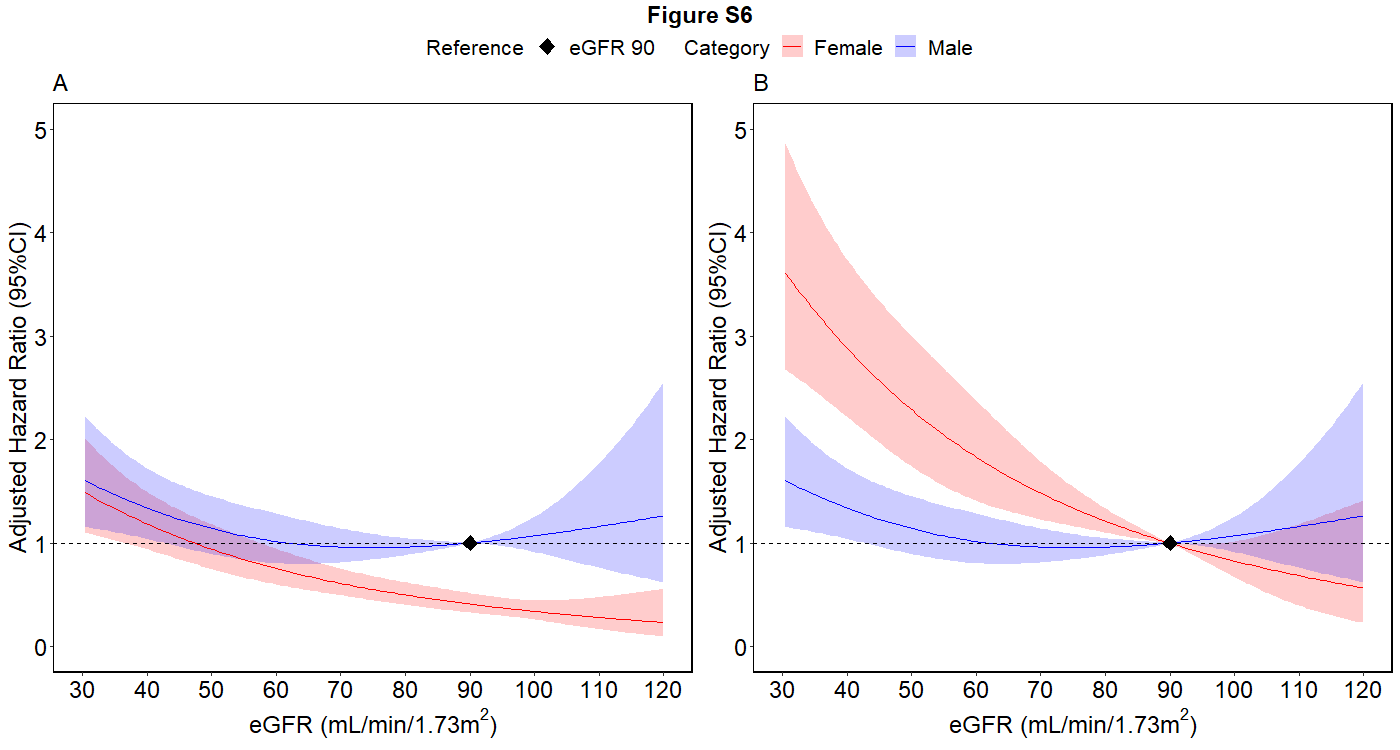


*eGFR × Gender interaction, p* <0.001

**A** Reference is eGFR 90 mL/min/1.73m^2^ in men. **B** Reference for men and women is eGFR 90 mL/min/1.73m^2^. Model: Elapsed time to CVD mortality = eGFR + gender + eGFR*gender + covariates. Covariates: age, albuminuria. Restrictive cubic spline was applied to eGFR with knots at 5^th^, 25^th^, 75^th^ and 95^th^ percentile. **Abbreviations:** eGFR, estimated glomerular filtration rate. CVD, cardiovascular disease.

**Supplemental Figure S7.** Cox regression model CVD mortality hazard curves adjusted for covariates characterizing 3-way interaction of eGFR, gender and age category (N=36556; years 2013-2019)


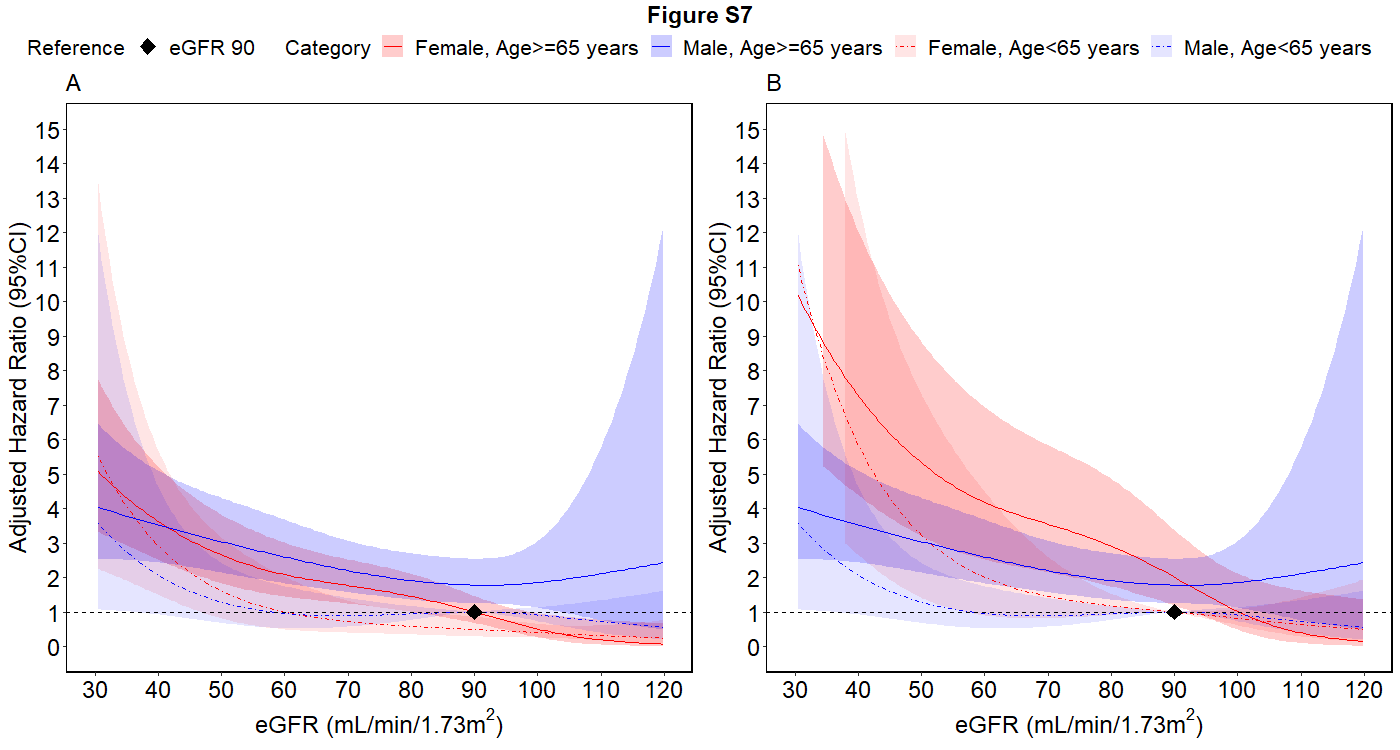


*eGFR × Age × Gender interaction, p* = 0.380

**A** Reference is eGFR 90 mL/min/1.73m^2^ in men aged <65 years. **B** Reference for men and women is eGFR 90 mL/min/1.73m^2^ aged <65 years. Model: Elapsed time to CVD mortality = eGFR + age + gender + eGFR*age + eGFR*gender + age*gender + eGFR*age*gender + covariates. Covariates: ethnicity, living in rental block, smoking, body mass index, lipid medications, hypertension, established CVD, albuminuria, systolic blood pressure, diastolic blood pressure, high-density lipoprotein, low-density lipoprotein, and triglycerides. Restrictive cubic spline was applied to eGFR with knots at 5^th^, 25^th^, 75^th^ and 95^th^ percentile. **Abbreviations:** eGFR, estimated glomerular filtration rate. CVD, cardiovascular disease.

**Supplemental Figure S8.** Cox regression simplified model all-cause mortality hazard curves adjusted for covariates characterizing 3-way interactions of eGFR, gender and age category (N=36556; years 2013-2019)


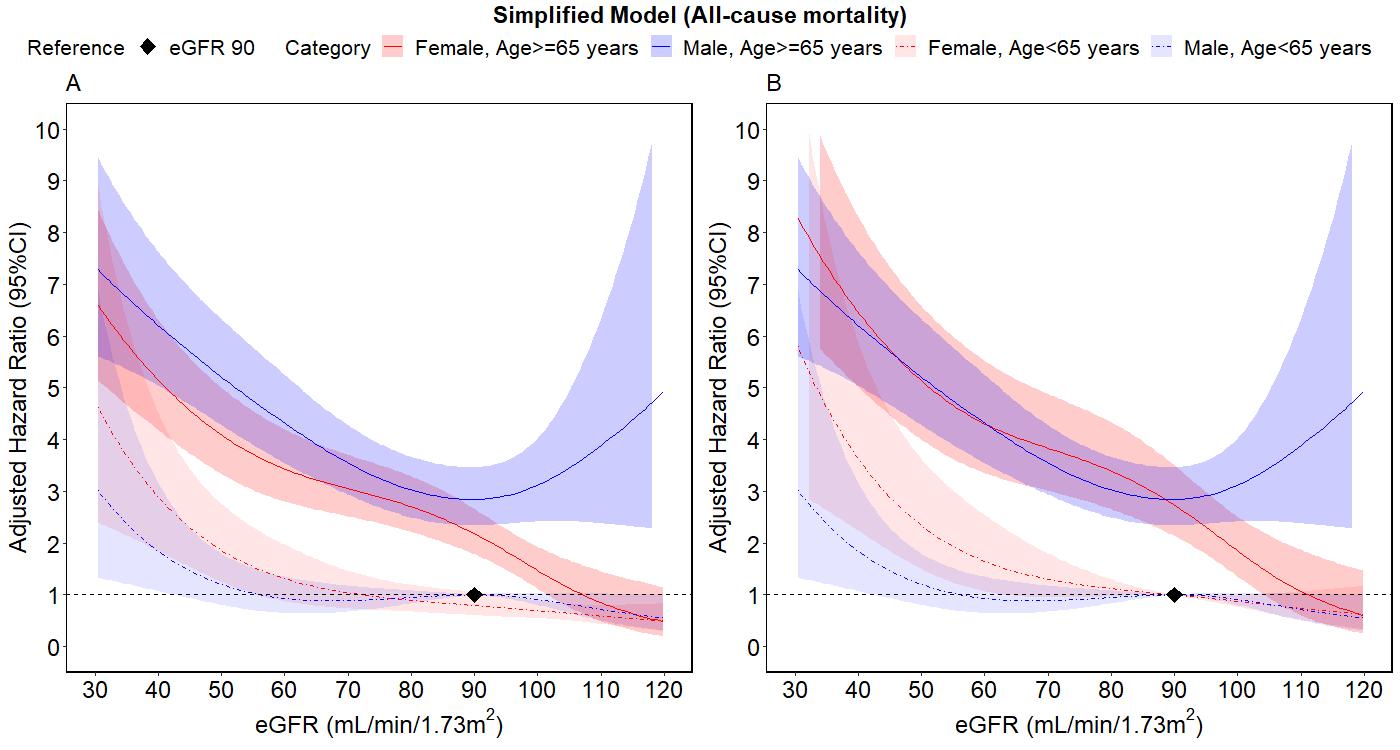


*eGFR × Age × Gender interaction, p*=0.022

**A** Reference is eGFR 90 mL/min/1.73m^2^ in men aged <65 years. **B** Reference for men and women is eGFR 90 mL/min/1.73m^2^ aged <65 years. Model: Elapsed time to all-cause mortality = eGFR + age + gender + eGFR*age + eGFR*gender + age*gender + eGFR*age*gender + covariates. Covariates: ethnicity, living in rental block, smoking, body mass index, lipid medications, established CVD and albuminuria. Restrictive cubic spline was applied to eGFR with knots at 5^th^, 25^th^, 75^th^ and 95^th^ percentile. **Abbreviations:** eGFR, estimated glomerular filtration rate. CVD, cardiovascular disease.

**Supplemental Figure S9.** Cox regression simplified model (excluding living in rental block) all-cause mortality hazard curves adjusted for covariates characterizing 3-way interactions of eGFR, gender and age category (N=36556; years 2013-2019)


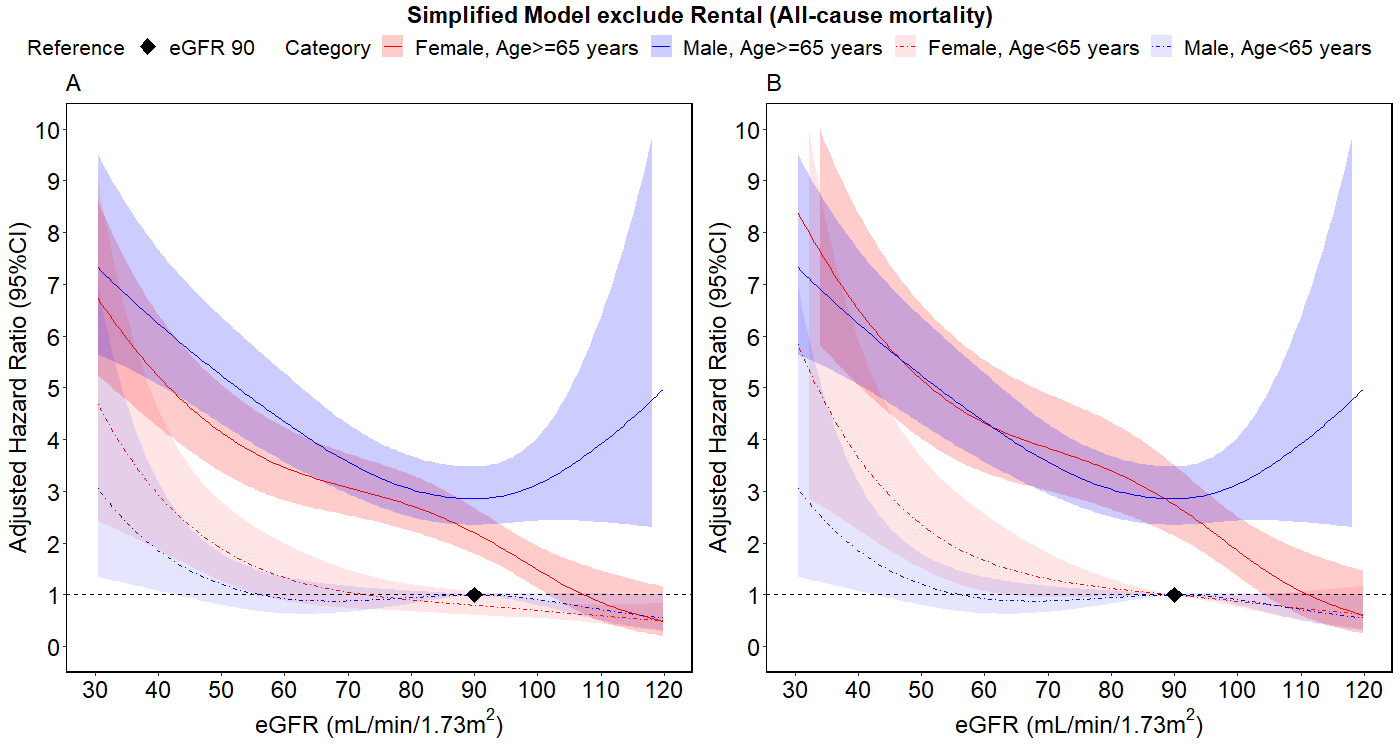


*eGFR × Age × Gender interaction, p*=0.022

**A** Reference is eGFR 90 mL/min/1.73m^2^ in men aged <65 years. **B** Reference for men and women is eGFR 90 mL/min/1.73m^2^ aged <65 years. Model: Elapsed time to all-cause mortality = eGFR + age + gender + eGFR*age + eGFR*gender + age*gender + eGFR*age*gender + covariates. Covariates: ethnicity, smoking, body mass index, lipid medications, established CVD, and albuminuria. Restrictive cubic spline was applied to eGFR with knots at 5^th^, 25^th^, 75^th^ and 95^th^ percentile. **Abbreviations:** eGFR, estimated glomerular filtration rate. CVD, cardiovascular disease.

**Supplemental Figure S10.** Cox regression simplified model CVD mortality hazard curves adjusted for covariates characterizing 3-way interactions of eGFR, gender and age category (N=36556; years 2013-2019)


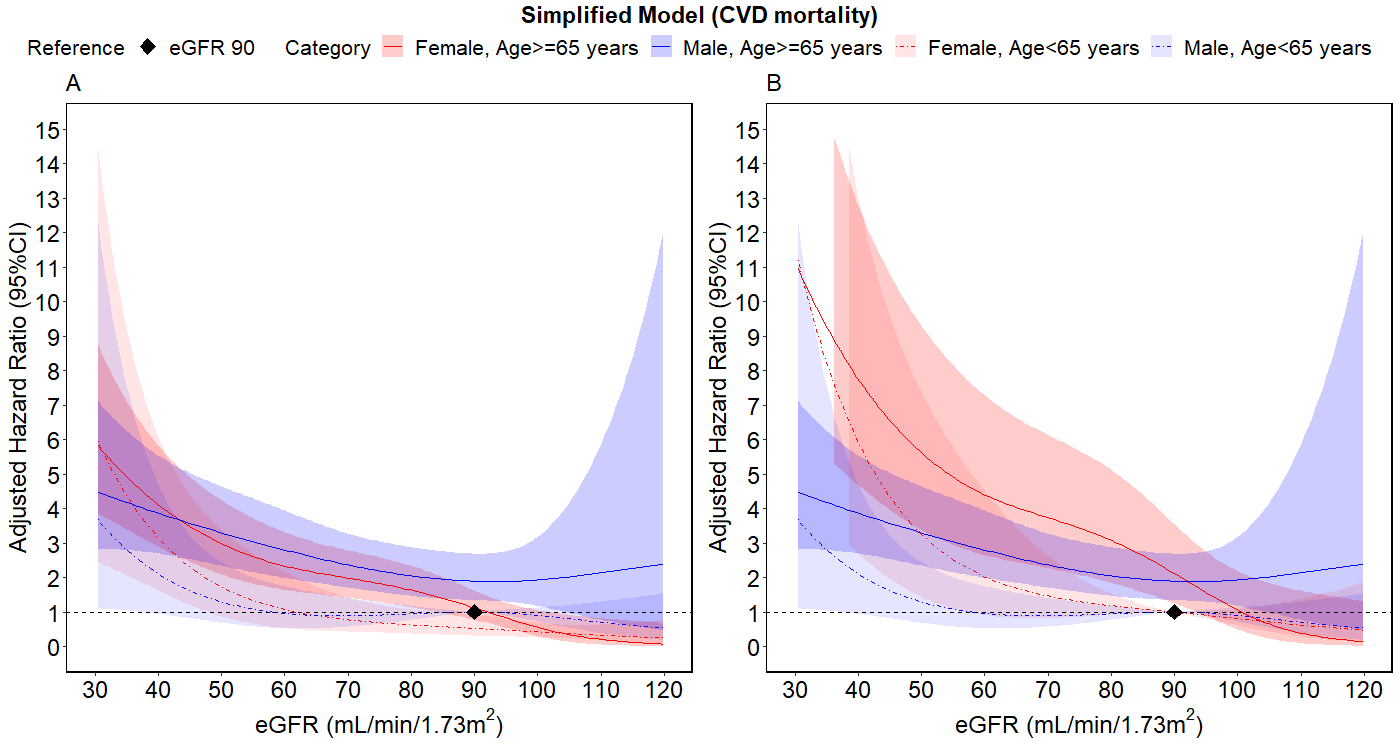


*eGFR × Age × Gender interaction, p*=0.379

**A** Reference is eGFR 90 mL/min/1.73m^2^ in men aged <65 years. **B** Reference for men and women is eGFR 90 mL/min/1.73m^2^ aged <65 years. Model: Elapsed time to all-cause mortality = eGFR + age + gender + eGFR*age + eGFR*gender + age*gender + eGFR*age*gender + covariates. Covariates: ethnicity, living in rental block, smoking, body mass index, lipid medications, established CVD and albuminuria. Restrictive cubic spline was applied to eGFR with knots at 5^th^, 25^th^, 75^th^ and 95^th^ percentile. **Abbreviations:** eGFR, estimated glomerular filtration rate. CVD, cardiovascular disease.

**Supplemental Figure S11.** Cox regression simplified model (excluding living in rental block) CVD mortality hazard curves adjusted for covariates characterizing 3-way interactions of eGFR, gender and age category (N=36556; years 2013-2019)


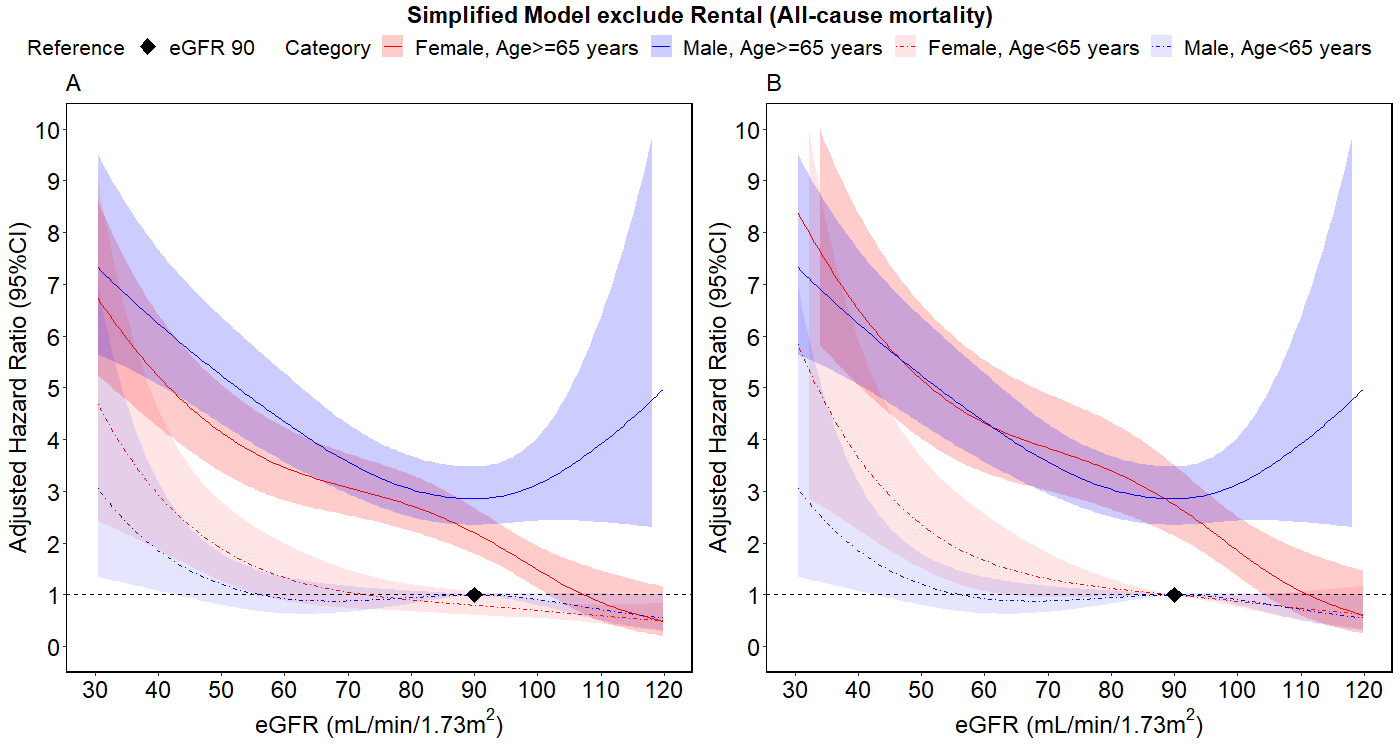


*eGFR × Age × Gender interaction, p*=0.376

**A** Reference is eGFR 90 mL/min/1.73m^2^ in men aged <65 years. **B** Reference for men and women is eGFR 90 mL/min/1.73m^2^ aged <65 years. Model: Elapsed time to all-cause mortality = eGFR + age + gender + eGFR*age + eGFR*gender + age*gender + eGFR*age*gender + covariates. Covariates: ethnicity, smoking, body mass index, lipid medications, established CVD, and albuminuria. Restrictive cubic spline was applied to eGFR with knots at 5^th^, 25^th^, 75^th^ and 95^th^ percentile. **Abbreviations:** eGFR, estimated glomerular filtration rate. CVD, cardiovascular disease.
